# In response to: Anatomy of ^18^F-GE180, a failed radioligand for the TSPO protein

**DOI:** 10.1007/s00259-020-04885-w

**Published:** 2020-06-10

**Authors:** Nathalie L. Albert, M. Unterrainer, L. Kaiser, M. Brendel, F. J. Vettermann, A. Holzgreve, P. Bartenstein

**Affiliations:** 1 Department of Nuclear Medicine, University Hospital, LMU Munich, Marchioninistr.15, 81377 Munich, Germany; 2 Department of Radiology, University Hospital, LMU Munich, Munich, Germany; 3 Athinoula A. Martinos Center for Biomedical Imaging, Department of Radiology, Massachusetts General Hospital, Harvard Medical School, Charlestown, MA USA

Dear Sir,

We read with interest the recently published letter by Zanotti-Fregonara et al. [1] that very critically discusses the characteristics of ^18^F-GE180 as a PET tracer for the 18-kDa mitochondrial translocator protein (TSPO) in human brain. This follows a previously published and rather similar letter and thus offers little information that is substantially new [2].

The main hypotheses of Zanotti-Fregonara et al. [1] are still that

1. The uptake of ^18^F-GE180 is not specific for TSPO and instead mainly reflects a broken and disrupted blood-brain barrier (BBB)
2. The sensitivity of the tracer must be low because differences in tracer binding cannot be detected between binding affinity types in vivo

The evidence that Zanotti-Fregonara et al. present in support of these hypotheses is not valid. From our point of view, the complete discreditation of a tracer is a harsh assessment and one that must be firmly based on scientifically conclusive evidence. Taking into consideration all published data as well as our own experience with the tracer, the arguments of the authors, although partially understandable, are not sufficient to support their general dismissal of ^18^F-GE180.

**Fig. 1:**
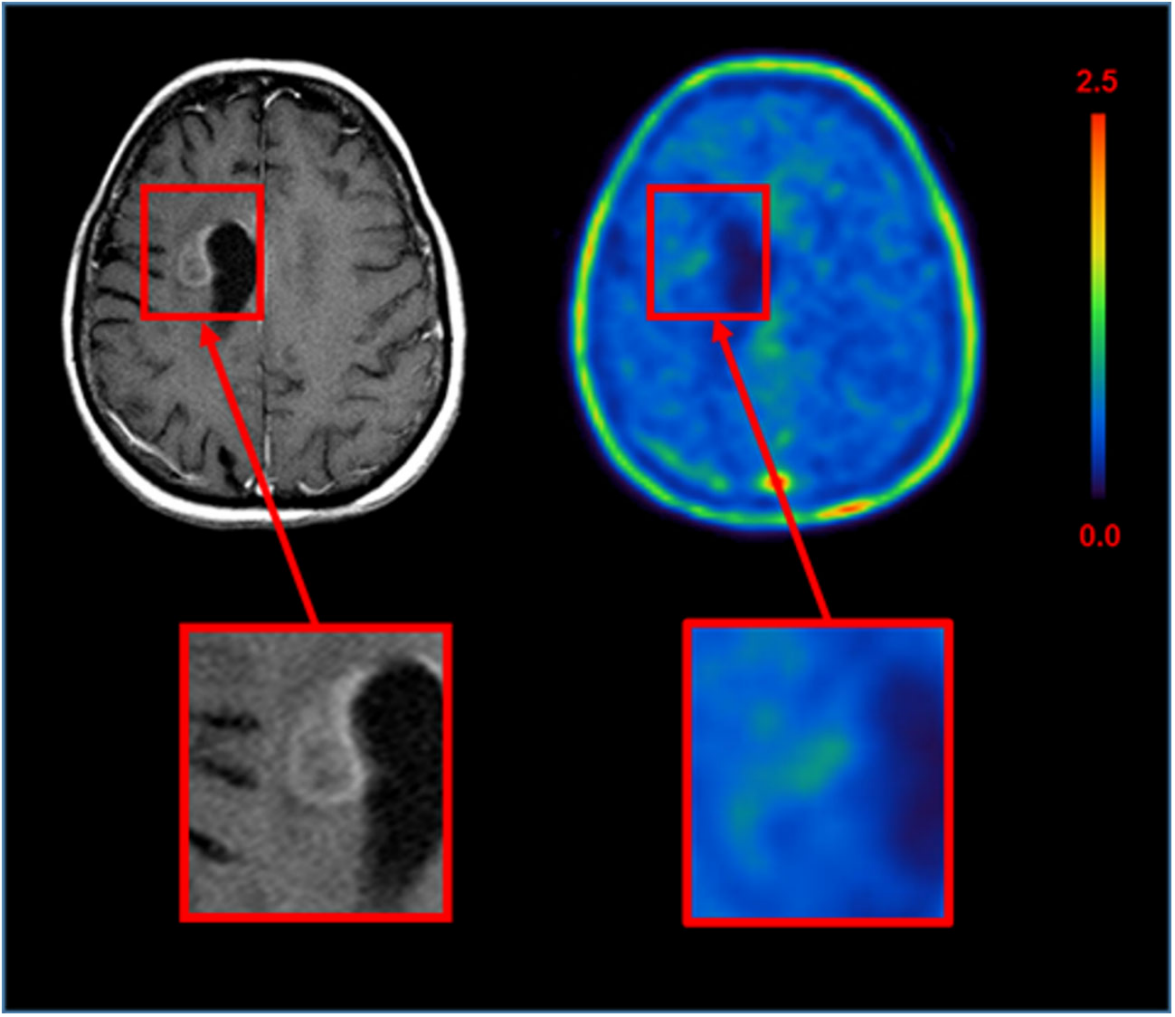
Example of a patient with a glioblastoma multiforme after radiotherapy: on MRI, there is a remaining contrast enhancement reflecting major BBB disruption; in the ^18^F-GE180 PET, no relevant corresponding tracer uptake can be found, showing that a mere macro-disruption of the BBB does not necessarily lead to a highly elevated, nonspecific tracer accumulation (PET image is SUV scaled)

**Fig. 2:**
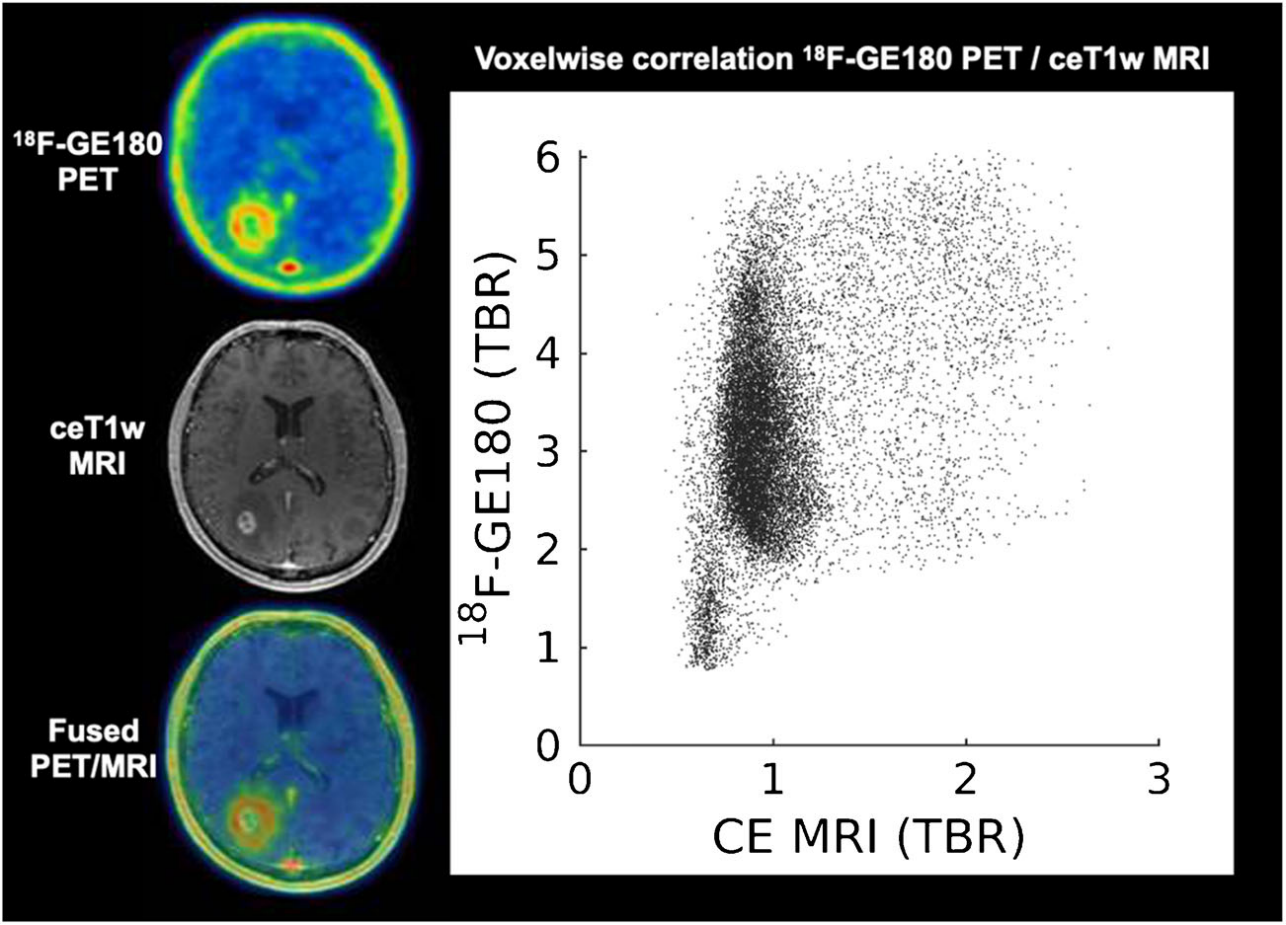
Example of a patient with a newly diagnosed glioblastoma—already the visual inspection shows that the maximal tracer signal can be found in the areas surrounding the contrast-enhancing ring lesion; the voxel-wise correlation of ^18^F-GE180 (tumour-to-background ratio (TBR); background assessed in 6 crescent-shaped regions-of-interest in the contralateral hemisphere according to [19]) and gadolinium signal intensity (TBR) [22] within all suspicious voxels on T2w MRI clearly reveals no significant association. To be more specific, the big cluster with contrast enhancement on background level (TBR ~ 1) shows increased uptake with a wide range of voxel values on ^18^F-GE180 PET. Moreover, even for voxels with contrast enhancement on MRI, no correlation with ^18^F-GE180 uptake can be identified

**Fig. 3:**
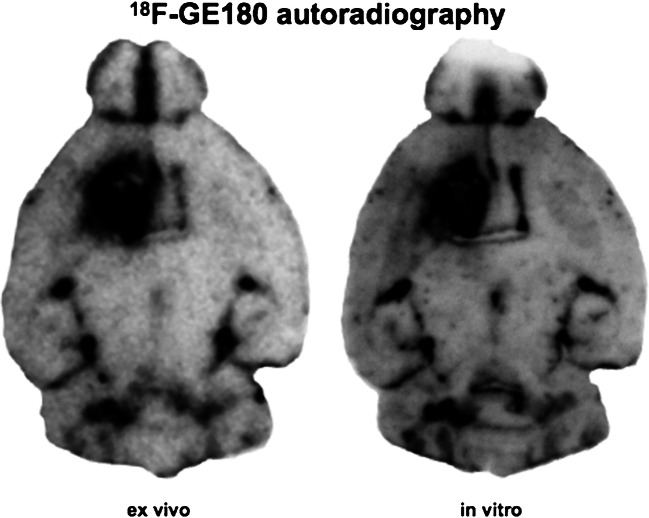
Example of a mouse glioblastoma with high tracer binding in the ex vivo autoradiography (left) performed following a ^18^F-GE180 PET scan and same signal pattern in the in vitro autoradiography (right). For the in vitro autoradiography, the tracer ^18^F-GE180 is applied directly to the slices (here even to the corresponding slice for optimal comparability) several days and weeks later. The tracer signal here is clearly not related to a blood-brain-barrier disruption

In our studies of glioma patients [3–5], we found that ^18^F-GE180 PET tracer uptake could be clearly visualized on the PET image in areas outside of the gadolinium enhancement area on MRI and even in gliomas without any visible contrast enhancement on MRI; Zanotti-Fregonara et al. argue that the BBB in these areas might be disrupted in terms of a microdisruption which is simply not depicted on MRI. We fully agree that the presence of an “MRI-invisible microdisruption” of the BBB in these cases cannot be ruled out. However, even if there was a microdisruption of the BBB, which can only be passed by the PET imaging probe and not by gadolinium, the hypothesis that the accumulation of ^18^F-GE180 beyond the BBB is merely nonspecific signal and is a speculation that is not firmly based on scientifically conclusive evidence. We claim that TSPO-expressing tissue beyond the BBB is required for the highly increased tracer signal found in our glioma studies. Our conclusion is based on several findings:

1. If a tracer signal reflects BBB breakdown only, one would expect to find an increased signal in all areas with disrupted BBB. In this context, Zanotti-Fregonara correctly stated that “the *direction* of the mismatch is important” and “If the gadolinium area is larger than that of ^18^F-GE180, one can plausibly argue for a dissociation between ^18^F-GE180 uptake and BBB breakdown.” [1]. In our earlier response letter [6], we presented an example of a patient with clear BBB breakdown (ring enhancing glioma lesion after radiotherapy), but without significant ^18^F-GE180 uptake in this area (see Fig. 1). This finding together with the intense tracer accumulation found in many other lesions without visible contrast enhancement in MRI makes it very unlikely that the cerebral uptake of ^18^F-GE180 is driven by the BBB disruption only. The authors appear to have disregarded this argument in their current letter [1]. The discrepancy between gadolinium enhancement and tracer signal *in the presented direction—*as suggested by Zanotti-Fregonara et al.*—*clearly points to a specific component necessary for sustained ^18^F-GE180 binding.
2. If the ^18^F-GE180 signal is primarily reflective of nonspecific tracer accumulation predominantly driven by disruption of the BBB, one would expect a correlation between the level of BBB disruption and ^18^F-GE180 signal intensity. However, there is in our experience no correlation between BBB disruption as assessed using contrast-enhanced MRI and the ^18^F-GE180 signal intensity (see Fig. 2 for an example).
3. Another argument for the specific binding of ^18^F-GE180 to TSPO is given by very similar binding patterns when comparing in vitro autoradiography with ex vivo autoradiography in animal models (Fig. 3).

Zanotti-Fregonara et al. insist on the importance of a correlation of in vivo ^18^F-GE180 uptake with histopathological analysis using stereotactic brain biopsies. We have an ongoing publicly funded study correlating ^18^F-GE180 uptake and histopathology in brain tumours exactly addressing this issue (German Research Foundation, FOR 2858; project A1 and A2). We evaluate here in-depth the correlation between TSPO expression and ^18^F-GE180 uptake in humans and hope to reproduce the promising results obtained in preclinical studies; e.g., Parhizkar et al. were able to demonstrate a strong correlation between the phagocytosis marker CD68 and the ^18^F-GE180 μPET signal in a transgenic amyloid mouse model [7]. Significant associations between ^18^F-GE180 PET and microglia immunohistochemistry were also proven for the more general microglia activation marker Iba1 in different amyloid mouse models [8, 9]. Considering this variety of evidence together with the minor impact of BBB leakage in amyloid mouse models, it seems very unlikely that a parallel BBB-related phenomenon confounds all observed associations of PET with gold standard assessments.

Zanotti-Fregonara et al. are of course correct in stating that the disruption of the BBB has an influence on the ^18^F-GE180 uptake, but this is a phenomenon that is more or less shared among all other TSPO tracers. To our knowledge, no publication has proven that the uptake of any other TSPO tracers e.g. is *not* influenced by the BBB disruption. Zanotti-Fregonara et al. themselves provide an example for the TSPO tracer ^11^C-ER176, which shows moderate uptake within the area of increased ^82^Rb-PET signal and contrast enhancement on MRI, but not beyond [1]. This tracer together with several other TSPO tracers has, compared with ^18^F-GE180, the disadvantage of exhibiting a very high fraction of metabolised tracer potentially contributing to tracer signal in regions with disrupted BBB. In contrast, ^18^F-GE180 has a favourably high parent fraction even at late time points [10, 11].

Furthermore, the statement by Zanotti-Fregonara et al. attributing the reported positive correlation of ^18^F-GE180 signal and the WHO grade in glioma [3] to increased levels of BBB disruption only is not correct. It may be true that the level of BBB disruption is—in many cases—higher in high-grade than in low-grade gliomas; however, up to 40% of gliomas without contrast enhancement on MRI are high-grade gliomas [12, 13]. TSPO expression itself is, independent of potential BBB disruption, also highly correlated with the WHO grade as shown by neuropathological studies [14]. Nonetheless, we agree with Zanotti-Fregonara et al. that the data on normal volunteers indicate that a certain basic microdisruption might be necessary for ^18^F-GE180 accumulation and therefore, TSPO-expression in an area completely without BBB-disruption might be underestimated by ^18^F-GE180 PET. However, as mentioned above, there is barely any clinical situation, in which brain pathologies (e.g. neuroinflammatory processes, gliomas, or their local co-existence) are not accompanied by at least a minor disruption of the BBB. Thus, this phenomenon cannot be ruled and is not of major practical relevance in investigating the diseased brain. It is of course highly desirable to understand in-depth the influence of BBB breakdown on ^18^F-GE180 uptake and similar tracers, e. g. by using adequate methodology including multi-modal PET and elaborate MRI analyses.

In their most recent letter, Zanotti-Fregonara et al. state: “The substantial role of BBB disruption in the uptake of ^18^F-GE180 is clearly shown in a recent pilot study in patients with ischemic lesions […]. The authors found that a significant part of the high uptake in the lesions is nonspecific and driven by BBB damage. Furthermore, up to 50% of the signal in nonischemic brain is due to vascular noise.” [1]. Keeping in mind that the signal fraction caused by vasculature has been reported to be about 20% for ^18^F-GE180 [11] (and not 50%) of the signal in healthy tissue at late time points (best representing distribution volumes), we do not support the above-drawn conclusions of Zanotti-Fregonara et al. A high vascular proportion of tissue signal of up to 50% is only true for the very early time frames (15–30 min post injection) used by Visi et al., which do not represent distribution volume [11]. Their hypothesis that high ^18^F-GE180 uptake in lesions is mainly non-specific is only based on the observation that a high uptake in one low-affinity binder (LAB) lesion was found in early static images, see [15]. Visi et al. observed otherwise a high correlation of lesion-to-reference ratios of the early ^18^F-GE180 signal with distribution volume ratios obtained using ^11^C-(R)-PK11195, indicating very similar in vivo behaviour [15]. This included the uptake in both observed LAB lesions. Due to the unclear susceptibility to TSPO polymorphism in vivo [10, 11, 16, 17], we believe that the high uptake in one LAB lesion does not prove non-specificity of the ^18^F-GE180 signal, but rather likely represents disease severity and TSPO expression level similar to ^11^C-(R)-PK11195.

With respect to the authors’ second claim that “the quality of the images is so poor that even a difference this large cannot be consistently detected” [1], we primarily refer to our previous reply letter [6]. The controversial results reported for ^18^F-GE180 uptake in healthy volunteers with different binding affinity status could be attributed to the low target expression in healthy tissue itself and the technical difficulties associated with ^18^F-GE180 quantification, see e. g. [17]. Moreover, one has to discuss to what extent the diagnostic quality of a tracer is limited by a very low signal of the tracer in healthy brain. A high target-to-background ratio allowing sensitive detection of TSPO expressing diseased tissue is the primary goal in a clinical setting. We believe that ^18^F-GE180 has suitable characteristics for that. It is quite obvious that specific tracer uptake is (besides the extensively discussed influence of BBB disruption) heavily influenced by the pathology itself, in such a way that potential differences induced by the binding affinity status in pathological lesions are very hard to detect; e.g., in patients with brain tumours, tumoural aggressiveness and inflammatory activity have a major effect on TSPO expression and will mask differences between the diverging binding affinity status at least for high- and medium-affinity binders, which represent the majority of the patients.

In summary, we reject the claim of Zanotti-Fregonara et al. that ^18^F-GE180 is a “failed radioligand”. Vice versa, we would like to clarify that ^18^F-GE180 cannot legitimately be called “superior” or an ideal radioligand in the light of the given limitations highlighted in the joint discussion [1, 2, 6]. We believe that a “perfect” radioligand targeting the TSPO does not (yet) exist—neither ^18^F-GE180 nor any other ligand so far—as every existing TSPO radioligand suffers drawbacks [18], which have to be weighed and thoughtfully considered during data interpretation. However, for beneficial routine clinical use, one has to compromise when dealing with the limitations of TSPO ligands. We believe—despite the discussed drawbacks of ^18^F-GE180—that imaging with ^18^F-GE180 can add comprehensive clinical information: First, we think that ^18^F-GE180-uptake in patients with multiple sclerosis and clinical worsening despite unchanged and non-enhancing lesions on MRI can indicate remaining disease activity [19, 20]—independent of the very exact contribution of the BBB on ^18^F-GE180 uptake. In these patients, standard imaging with contrast-enhanced MRI is less sensitive in assessing the change of the clinical situation. Second—also independent of the very exact contribution of the BBB on ^18^F-GE180 uptake—we believe that imaging with ^18^F-GE180 can significantly add clinical information in brain tumour patients by delineating the tumour extent beyond contrast enhancement on MRI [3–5, 21] and, in future, probably by depicting in more detail, compared with labelled amino acids, the tumour characteristics. Therefore, in summary, we believe that despite the repeated articulation of ^18^F-GE180 PET drawbacks, its use can significantly contribute to clinical issues that thus far cannot be resolved with standard imaging alone.

Finally, we wanted to strongly emphasize our appreciation of Dr. Owen’s previous and current work. We do not doubt Dr. Owen’s theses in personal communications at all, as probably inadvertently indicated by Zanotti-Fregonara et al. [1]. By contrast, we rather wanted to encourage the authors to further obtain and publish data on in vitro affinity to TSPO polymorphism in order to foster fruitful exchange and help to solve the common controversial and important issues.
